# Investigating Real-World Benefits of High-Frequency Gain in Bone-Anchored Users with Ecological Momentary Assessment and Real-Time Data Logging

**DOI:** 10.3390/jcm10173923

**Published:** 2021-08-30

**Authors:** Arjan J. Bosman, Jeppe Høy Christensen, Tove Rosenbom, François Patou, Arno Janssen, Myrthe K. S. Hol

**Affiliations:** 1 Department of Otorhinolaryngology, Radboud University Medical Center, P.O. Box 9101, NL-6500 HB Nijmegen, The Netherlands; arno.janssen@radboudumc.nl (A.J.); myrthe.hol@radboudumc.nl (M.K.S.H.); 2 Eriksholm Research Centre, Oticon A/S, Rørtangvej 20, DK-3070 Helsingør, Denmark; jych@eriksholm.com; 3 Oticon Medical, Kongebakken 9, DK-2765 Copenhagen, Denmark; tvro@oticonmedical.com (T.R.); fpat@oticonmedical.com (F.P.); 4 Department of Otorhinolaryngology, University Medical Center Groningen, P.O. Box 30001, NL-9700 RB Groningen, The Netherlands

**Keywords:** bone conduction device (BCD), data logging, ecological momentary assessment (EMA), linear mixed-effects regression, hearing loss

## Abstract

Purpose: To compare listening ability (speech reception thresholds) and real-life listening experience in users with a percutaneous bone conduction device (BCD) with two listening programs differing only in high-frequency gain. In situ real-life experiences were recorded with ecological momentary assessment (EMA) techniques combined with real-time acoustical data logging and standard retrospective questionnaires. Methods: Nineteen experienced BCD users participated in this study. They all used a Ponto 4 BCD from Oticon Medical during a 4-week trial period. Environmental data and device parameters (i.e., device usage and volume control) were logged in real-time on an iPhone via a custom iOS research app. At the end of the trial period, subjects filled in APHAB, SSQ, and preference questionnaires. Listening abilities with the two programs were evaluated with speech reception threshold tests. Results: The APHAB and SSQ questionnaires did not reveal any differences between the two listening programs. The EMAs revealed group-level effects, indicating that in speech and noisy listening environments, subjects preferred the default listening program, and found the program with additional high-frequency gain too loud. This finding was corroborated by the volume log—subjects avoided the higher volume control setting and reacted more to changes in environmental sound pressure levels when using the high-frequency gain program. Finally, day-to-day changes in EMAs revealed acclimatization effects in the listening experience for ratings of “sound quality” and “program suitability” of the BCD, but not for ratings of “loudness perception” and “speech understanding”. The acclimatization effect did not differ among the listening programs. Conclusion: Adding custom high-frequency amplification to the BCD target-gain prescription improves speech reception in laboratory tests under quiet conditions, but results in poorer real-life listening experiences due to loudness.

## 1. Introduction

A percutaneous bone conduction device (BCD) is a viable solution for patients with a conductive or mixed hearing loss (HL) [1,2,3], or with single-sided deafness [4]. A BCD comprises of a sound processor fixated with a skin-penetrating abutment on a titanium implant (fixture) anchored in the skull. The sound processor converts sounds into mechanical vibrations that are transferred through abutment, implant, and skull to the cochlea.

By default, the effective gain of a BCD, defined as the bone conduction threshold minus the aided threshold [5], rarely results in a complete closure of the air-bone gap. For example, in patients with conductive losses and average BC thresholds of 10 or 20 dB HL, an effective gain was found of −6 dB [6] or −7 dB [7], respectively. These findings were corroborated by [5], showing negative effective gain values in patients with average bone conduction thresholds at 1, 2, and 4 kHz, up to 25 dB HL. It is thus worth investigating whether increasing gain relative to a default BCD target-gain prescription is beneficial to the listener. Given the small headroom of BCD devices for frequencies below 1 kHz [8], and the relatively small contribution of low frequencies to speech intelligibility [9], we decided to increase gain only for frequencies above 1 kHz.

The benefits of percutaneous BCDs have been clearly demonstrated using speech intelligibility measurements [10,11], and retrospective questionnaires such as the Speech Spatial Qualities (SSQ) [11,12] or the Abbreviated Profile of Hearing Aid Benefit (APHAB) [10,13,14,15]. However, independent data on real-life patient outcomes and limitations beyond listening tests in simulated real-world environments are lacking [16].

Methods of evaluating listening benefits from BCDs are currently limited to laboratory settings or retrospective reports, which hinders investigations into real-life and context-specific benefits. Well-controlled listening tests do provide reliable data, but often lack validity, as actual real-life listening conditions can be vastly different from those inside the laboratory. Typically, laboratory testing of hearing abilities reaches its ceiling at negative or close to zero SNRs [17,18,19], whereas real-world SNRs are rarely negative [20,21,22,23]. For example, Wu et al. [22] report that 63% of their recorded real-world SNRs were between 4- and 14-dB; Smeds et al. [20] reported a mode in the distribution of real-world SNRs between 2- and 6-dB. In contrast to laboratory-based listening tests, self-reports and questionnaires, such as the aforementioned SSQ and APHAB, have high face validity [24,25]. However, with retrospective self-reports, subjects need to memorize their experiences with certain listening situations, dating back days, weeks, or even months. This delayed recall may bias overall judgements, as subjects tend to put more emphasis on recent experiences [26]. Additionally, when answering questionnaires, listeners need to aggregate and generalize experiences across various listening situations. Detailed contextual information of their experiences with specific listening situations, i.e., the ‘auditory ecology’ [27], will get lost. Therefore, both listening tests and self-reports may not appropriately reflect real-world performance [24,28], and the retrospective nature of questionnaires prevents assessments of day-to-day changes (i.e., acclimatization) in the listening experience [29].

An alternative to using traditional self-reports is the sampling of real-world experiences in situ, referred to as experience sampling or ecological momentary assessment (EMA) [30]. With the EMA approach, listener experiences are captured on a wearable device, e.g., a smartphone, in real time. The EMA methodology has been shown to be feasible for hearing aid evaluation [31] in exploring daily effects of tinnitus [32], exploring benefits from hearing aid noise management [33], and characterizing hearing problems of hearing aid users in everyday life [26]. In parallel to the EMA, selected parameters of the ambient acoustic environment, i.e., the ‘soundscape’, can be logged in real-time with the smartphone-connected hearing device. The combination of listener experiences and acoustical data provides insights into listener-specific problems in daily life [34,35]. Although several studies have reported on everyday listening experiences of hearing aid users [26,31], only a few previous studies have employed statistical modelling of EMA responses with real-time acoustical data [36,37,38] in order to identify targeted context-specific benefits.

The aim of this study is to evaluate whether a group of unilaterally fitted BCD users will benefit from custom high-frequency amplification, in addition to a standard prescription, under both real-life and laboratory conditions. Specifically, two listening programs differing only in high-frequency gain above 1.1 kHz are contrasted, and context-specific real-life outcomes are investigated with statistical modeling of in situ subjective listening experiences (EMAs), with real-time data logging of the ambient acoustic environment. The real-life outcomes are compared to traditional speech reception tests and retrospective questionnaires about listening experiences.

In supporting analyses, data logs of BCD volume control and day-to-day changes in EMAs are assessed. The latter is important for understanding acclimatization effects [29], by establishing how subjects’ listening experience changes with days of use, while the former might provide insights into subjects’ behavioral patterns. Specifically, while the target-gain prescription is fixed among the subjects, volume control usage across the trial period might reveal individual needs for additional and personalized fine-tuning.

## 2. Methods and Materials

### 2.1. Subjects

This study was designed to include twenty experienced BCD users, all with an air-bone gap of at least 20 dB and BC thresholds on the fitted side better than 45 dB HL. While we originally aimed to include 20 subjects, due to COVID-19 ‘lockdown’ measures we closed this study after including 19 subjects. At their first visit, all subjects gave their informed consent to participate in the study. Subject characteristics are shown in Table 1. The group was comprised of 8 females and 11 males with a median age of 47 years (range: 19–72 years). Seventeen subjects had about 5 years of experience with an earlier Ponto BCD (Oticon Medical, Askim, Sweden), whereas subjects 8 and 12 had 2 and 4 months of experience, respectively. The average bone conduction threshold at the fitted side was 20.9 dB HL (range: 3.8–36.3 dB HL).

Ten subjects (1, 3, 4, 8, 9, 10, 11, 14, 15, 18) had a bilateral conductive or mixed HL due to chronic suppurative otitis media (CSOM), and two subjects (5, 13) had a bilateral conductive loss due to congenital atresia. Five subjects had a unilateral HL due to CSOM (2, 6, 7, 12, 17). On the contralateral side, two subjects had normal hearing (2, 12), two subjects had a sensorineural HL (6, 7), and one subject had no contralateral hearing (17). Subjects 16 and 19 had a unilateral HL due to congenital atresia, with normal hearing on the contralateral side.

### 2.2. Devices

All subjects were fitted with a new Ponto 4 BCD with two listening programs. At the first visit, pure-tone thresholds were measured with a clinical audiometer (Interacoustics Equinox, Assens, Denmark) using TDH-39 headphones and a B-71 bone conductor, and BC in situ thresholds were measured with the BCD. From the BCD threshold, a default listening program (P_d_) was created with the Genie Medical fitting software (Oticon Medical). This default program was copied to a custom program (P_c_), and its gain settings were modified using an Excel worksheet implementing the following rule:

(1) $$\mathrm{Custom}\;\mathrm{gain}=\begin{array}{c} G_{0}+2\mathrm{dB}+0.33*\mathrm{BC}\;\mathrm{threshold}\;\mathrm{for}\;\mathrm{frequencies}=1.1\;\mathrm{kHz}\\ G_{0}+5\mathrm{dB}+0.33*\mathrm{BC}\;\mathrm{threshold}\;\mathrm{for}\;\mathrm{frequencies}>1.1\;\mathrm{kHz} \end{array}$$

with G_0_ = default gain for a 0-dB HL BC threshold.

Thus, both programs had identical gain settings for frequencies below 1.1 kHz, corresponding to the prescription of the Genie Medical fitting program. In P_d_ this prescription was also used for the higher frequencies, whereas in P_c_ an input-independent (linear) gain prescription (Equation (1)) was used with a modified third-gain rationale. The programs P_c_ and P_d_ were randomly assigned as listening program 1 or 2 in the BCD.

The rationale in P_c_ resulted in at least 5 dB more gain for frequencies above 1.1 kHz than with P_d_, across all subjects. The average 1–4 kHz gain for input levels of 50, 65, and 80 dB SPL was 3.0, 7.2, and 11.7 dB higher for P_c_ than for P_d_, respectively.

### 2.3. Experimental Procedure

#### 2.3.1. EMA Field Test

During a 4-week trial period (actual days of testing varied, see Table 2), subjects used the Ponto 4 BCD connected via Bluetooth to an iPhone, with an iOS research app from Oticon Medical for changing the listening program and volume (using 2.5-dB steps), for logging their in situ EMA responses, and for storing acoustical data of the momentary listening environment. Subjects without an iPhone received a loaner iPhone for the trial period.

For the trial period, P_c_ was randomly assigned as program 1 or 2. Subjects were encouraged to change the listening program as often as practically feasible. Upon changing the listening program, users were prompted to respond to a five-question EMA on a continuous scale: (1) How do you rate loudness? (2) How do you rate sound quality? (3) How suitable is this program for this environment? (4) Did you listen to speech? If yes: (5) How do you rate speech intelligibility? Henceforward, the four EMA questions are referred to as “Sound quality”, “Program suitability”, “Loudness”, and “Speech understanding”, respectively.

#### 2.3.2. Follow-Up Laboratory Tests and Questionnaires

At the second visit (i.e., end of the trial), speech perception in quiet and noisy environments was measured for both listening programs. Speech-in-quiet was measured with NVA-monosyllabic word lists, comprising 11 consonant-vocal-consonant syllables per sublist [39]. The sublists were presented at 40, 50, 60, and 70 dB SPL in free field with speech from the front. No contralateral masking was applied, mimicking real-life device use. The speech reception threshold (SRT) was calculated from the performance-intensity curve by interpolating levels that yielded scores just below and above 50%. Speech perception in a noisy environment was measured with the sentence material of Plomp and Mimpen [40], with both speech and 65 dBA noise presented from the front. The SRT in the noisy environment was measured with an adaptive ‘one up, one down’ procedure with 2-dB steps, as suggested by [40].

In addition, overall experiences with both programs were probed with “The Abbreviated Profile of Hearing Aid Benefit” (APHAB) [41] and “The Speech, Spatial and Qualities of Hearing Scale” (SSQ) [42] questionnaires, as well as with a proprietary preference questionnaire, using five-point Likert scales for clarity, loudness, and overall preferred listening program. The APHAB [41] is a 24-item questionnaire for assessing hearing problems in daily life. The frequency of problems listeners had with communication or loud noises were scored on four subscales: ease of communication (EC), background noise (BN), reverberation (RV), and aversiveness of loud sounds (AV). The SSQ [42] is a 49-item self-assessment of hearing disability in situations typical of real life. It comprises 14 items on speech hearing (speech), 17 items on spatial hearing (spatial), and 18 items on quality of sound (quality). All questionnaires were administered at the second session for the aided condition in order to obtain a direct comparison of both listening programs. At the end of this visit, the experimenter retrieved all logged data from the iPhone, comprising both the acoustical data by the BCD and the subject’s EMAs for further off-line analysis.

All users decided to keep their BCD. The BCD was programmed according to the subject’s preferences, the research app was removed from the iPhone, and the standard Oticon ON app for controlling the device and Bluetooth sound streaming was installed on the subject’s smartphone.

### 2.4. Data Logging and Data Pre-Processing

A time-stamped sound pressure level (SPL) and signal-to-noise (SNR) estimate, together with an acoustic analysis classifying the listening environment as ‘Quiet’, ‘Noise’, Speech’, and ‘Speech in Noise’ (soundscape), were logged and stored on the iPhone every 80 s of BCD wear time as long as the iPhone had a Bluetooth connection with the BCD. All measures are available in standard commercial Ponto 4 BCDs, and the acoustic data are similar to those logged by standard Oticon Op S behind-the-ear hearing aids (see Christensen et al., 2019 and Christensen et al., 2021 [23,43] for more details about the acoustic data). In brief, momentary sound input was recorded by two calibrated microphones situated in the hearing device and sensitive to sound across a frequency range of 0–10 kHz. The SPL is the level output estimate from a low-pass infinite impulse response filter with a time constant of 63 ms. The SNR was computed as the difference between a bottom tracker (noise floor), which was implemented with a dynamic attack time of 1–5 s and a release time of 30 ms, and the SPL (for an illustration, see Figure 10.3 in [44]).

To obtain robust estimates of the ambient sound environment associated with self-reported listening experiences and BCD usage, each logged EMA and volume change (irrespective of listening program) were associated with the average of the three prior logged acoustic data samples. Thus, short-term sound exposure was associated to changes in both volume and ratings of the two listening programs. Note that since the BCD automatically sets the volume to 0 on reset and program change, all logged volume changes to level 0 were excluded from further analysis.

Observations were predominantly classified as belonging to either Quiet or Speech soundscapes (37.6% and 36.9%, respectively), with observations in Speech in Noise and Noise being sparse (18.3% and 7.3%, respectively). To increase statistical power, and given the high degree of overlap between Speech in Noise and Noise in terms of other acoustical characteristics [23], we collapsed these two soundscapes prior to further analysis (henceforward called Noisy Speech or Noise).

### 2.5. Statistical Analysis

We applied linear mixed-effect (LME) modelling to associate EMA ratings with days of BCD usage, to associate ambient sound levels (SNR and SPL) with volume adjustments, and to predict EMA ratings based on soundscape data and listening program. In addition, random effects were included to adjust the models for contextual confounds. For the first case, inter-individual variability in offsets and slopes were adjusted for by including a random term for subject ID. For the second case, the random effect structure was expanded to also include offsets due to time (i.e., hour of the day). Lastly, for the third case, the random effects structure additionally included days of usage offsets (i.e., acclimatization effect), and offsets due to volume setting.

The listening program and soundscape categorical predictors were contrasted against baseline conditions P_d_ and Quiet, respectively, and LME models were compared using likelihood-ratio tests to ensure optimal model fit.

We used *t*-tests or Kruskal–Wallis and Wilcoxon signed-rank tests (when normality could not be assumed) for additional paired or un-paired comparisons, and all statistical analysis were performed in R (version 3.6.1, “The R Foundation for Statistical Computing”, Vienna, Austria), with packages “nlme” for LME modelling, “stargazer” for regression coefficient statistical evaluation, and “coin” for Wilcoxon signed-rank tests.

## 3. Results

### 3.1. Standard Measures of Hearing Performance

**Figure 1:**
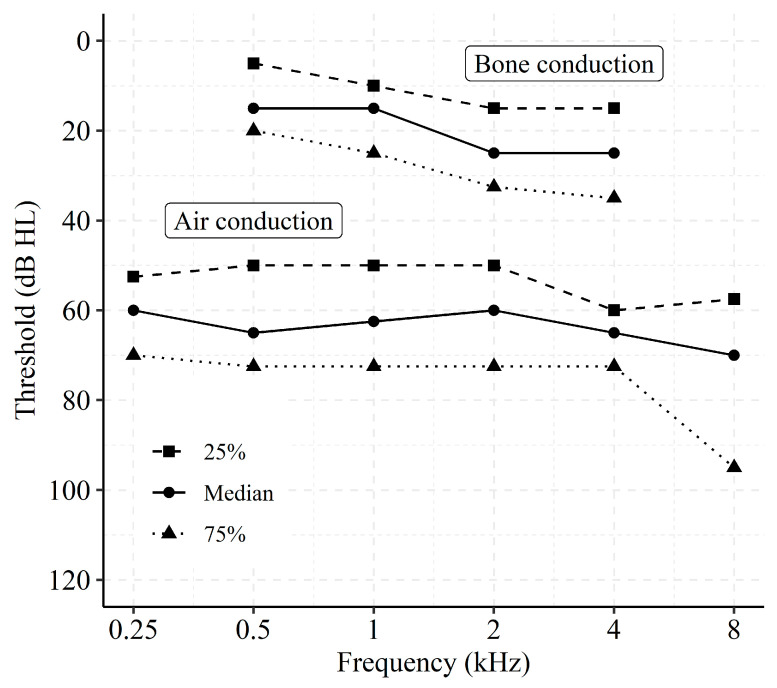
Distribution of air conduction and masked bone conduction thresholds at the fitted side expressed in 25%, 50%, and 75% percentiles for the 19 subjects.

Figure 1 shows the distribution of air conduction thresholds and masked bone conduction thresholds for the fitted side, expressed in 25%, 50%, and 75% percentiles. Averaged across all subjects, the pure-tone average threshold at 0.5, 1, 2, and 4 kHz was 63.4 dB HL for air conduction and 20.9 dB HL for bone conduction.

At the second visit, speech-in-quiet was measured with frontal speech for both listening programs with NVA monosyllables [39]. For the default program, the average SRT of 39.1 dB SPL (±1.4 dB, mean standard error) was significantly higher than the SRT of 36.9 ± 1.3 dB SPL for the custom program (paired *t*-test; *t* = 2.86, df = 18; *p* = 0.01). The signal-to-noise ratios for sentences [39] with frontal speech and 65 dBA frontal noise were −4.2 dB and −3.9 dB for the default and custom programs, respectively. This difference was not significant (paired *t*-test; *t* = 1.32, df = 18; *p* > 0.05).

At the second visit, subjects filled in APHAB and SSQ questionnaires for the aided conditions. Mean scores of the APHAB questionnaire for the domains ease of communication (EC), background noise (BN), reverberation (RV), and aversiveness of loud sounds (AV) were 21.2 ± 2.8, 39.4 ± 3.6, 32.0 ± 3.6, and 39.1 ± 5.3 for the default program and 23.7 ± 4.3, 42.1 ± 4.6, 32.5 ± 4.2, and 44.9 ± 6.0 for the custom program, respectively. For the SSQ questionnaire, mean scores for speech, spatial, and sound quality were 6.4 ± 0.3, 5.1 ± 0.6, and 7.1 ± 0.3 for the default program and 6.2 ± 0,4, 5.2 ± 0.5, and 7.0 ± 0.3 for the custom program, respectively. The APHAB and SSQ scores were not significantly different for the two listening programs (paired *t*-test; APHAB EC: *t* = 0.59, BN: *t* = 0.70, RV: *t* = 0.16, AV *t* = 2.11; SSQ Speech *t* = 1.53, Spatial *t* = 0.30, Quality *t* = 1.29; df = 16; *p* > 0.05).

Data from a retrospective questionnaire using five-point Likert scales for the overall preferred listening program revealed that 9 out of 18 subjects (data missing from one subject) preferred the default program, 6 preferred the custom program, and 3 did not have a preference.

### 3.2. Data Logging and In Situ Reports

The total amount of logged data (usage, acoustic data, and EMAs) per subject is summarized in Table 2. On average, data were logged for approximately 50% of the total BCD up-time (see Table 2, right column). Both the amount of data logging and the number of EMAs differed for the two listening programs, but the differences were not significant (Wilcoxon signed-rank tests; z = −1.65, *p* = 0.10, and z = −1.75, *p* = 0.08, respectively).

**Figure 2:**
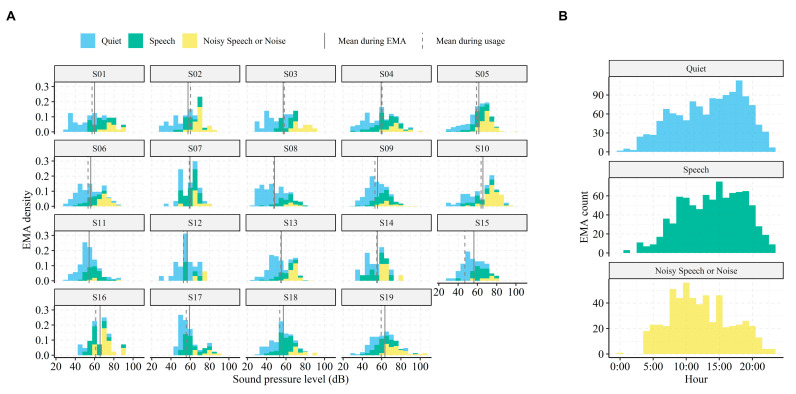
Data logging summary. (**A**) Density distributions of the sound pressure levels during Ecological Momentary Assessment (EMA) submissions per subject (panels) and separated by soundscape (colors, stacked). The density (vertical axis) represents the proportion of EMAs submitted within levels of SPL binned by 5 dB. The vertical lines indicate the mean SPL logged during EMA (solid line) and during normal usage (i.e., usage when not performing EMA; dashed line). (**B**) Histograms of all submitted EMAs per hour (pooled among all subjects) and separated by soundscape. Note the different scaling of the vertical axis on B.

We also investigated when and in which contexts EMAs were submitted. Figure 2A shows density plots of the SPLs encountered by each subject when performing EMAs. It shows that subjects varied in their auditory ecology—that is, some subjects were exposed to challenging and loud environments (e.g., subject 5 and 10), whereas others predominantly submitted EMAs in quiet conditions (e.g., subject 8 and 12). Across all subjects, the 25, 50, and 75% percentiles of encountered SNRs were 3.2, 7.5, and 14.1 dB when performing EMAs, and 1.5, 4.3, and 10.4 dB during normal usage times, respectively. In addition, most EMAs were performed in Quiet and Speech environments (see Figure 2B and Table 2), and those EMAs were skewed towards later times of the day (afternoon/early evening). In contrast, EMAs submitted in more challenging listening conditions were predominantly made around morning/noon. These patterns align well with what would be expected from normal device usage; that is, the BCD were predominantly used in Quiet and Speech environments (36% and 38% of total use time—see Table 2). A strong correlation between the relative amount of submitted EMAs and relative usage per soundscape indicate that, indeed, subjects in the current study completed EMAs in proportion to the time spent in these environments (Pearson’s product-moment correlation, *t*(55) = 7.45, *r* = 0.71, *p* < 0.001). Thus, the listening environments associated with EMAs were comparable to the listening environments during periods without EMAs.

**Figure 3:**
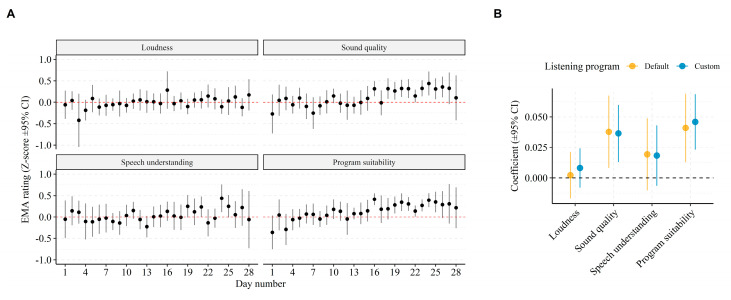
Acclimatization effect. (**A**) Grand average EMA ratings per day since field study start. The EMA ratings were individually z-score transformed prior to averaging to adjust for inter-individual variance. Red horizontal line at y = 0 indicates the overall mean rating. (**B**) Regression coefficients for predicting change in EMA ratings by days since field study start. The coefficients are computed by LME models adjusted for random slopes and intercepts by subjects.

Lastly, since all subjects were new to the Ponto 4 BCD, we expect a certain acclimatization to the BCD, as has been reported for new users of BTE hearing aids [29]. The grand average EMA rating for “Sound quality” was 5.8 (SD = 2.0) on the very first day of testing. This number increased to 7.11 (SD = 1.8) on the last day of testing, representing a 22.6% increase due to acclimatization. Figure 3A shows average EMA ratings per question and per day since day one of the study, corrected for inter-individual variance in EMA scores (i.e., z-score transformed). Notably, ratings of “Sound quality” and “Program suitability” increased beginning around 17 days of use. We quantified the acclimatization effect by regressing (using LME modelling) EMA ratings for each question with number of days from day one of the study and allowed for random slopes and offsets per subject. The resulting regression coefficients (shown in Figure 3B) indicate significantly increasing ratings for “Sound quality” (P_d_: *β* = 0.037, 95% CI = (0.008–0.067), *p =* 0.013; P_c_: *β* = 0.036, 95% CI = (0.013–0.060), *p =* 0.002), and “Program suitability” (P_d_: *β* = 0.041, 95% CI = (0.013–0.070), *p* = 0.004; P_c_: *β* = 0.046, 95% CI = (0.023–0.069), *p <* 0.001) with days of use. This effect did not differ significantly between the listening programs.

#### Comparing In Situ with Retrospective Preference Reports

In situ EMAs for each subject and listening program were averaged across time and EMA questions to represent “overall in situ preferences” (i.e., thereby disregarding specific listening conditions). In this respect, the overall preference refers to the average rating of “Sound quality”, “Speech understanding”, and “Program suitability” when conversation was indicated, and otherwise to the average rating of only “Sound quality” and “Program suitability” when subjects indicated that no conversation was taking place.

**Figure 4:**
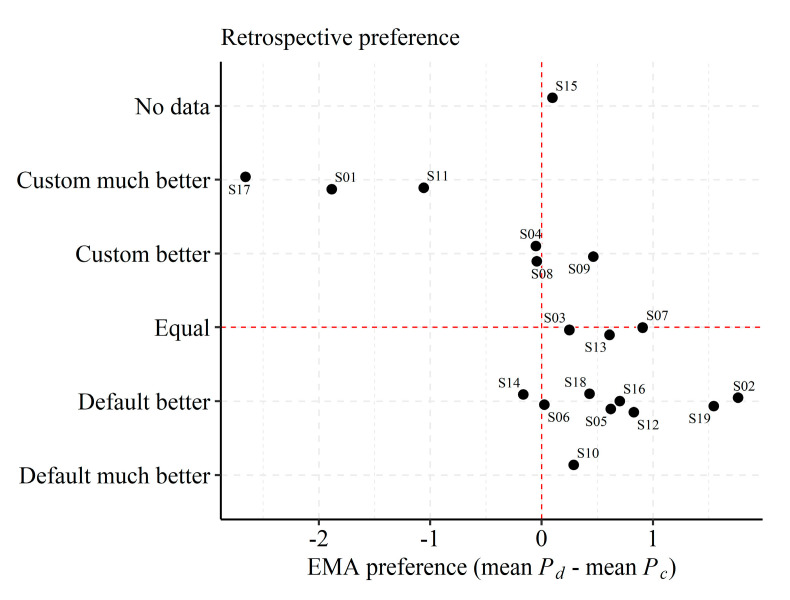
Retrospective preference against in situ EMA preference. EMA preference is represented by the difference in overall in situ rating of the default (P_d_) and custom (P_c_) listening program estimated from EMA responses. A positive x-value indicates a preference for P_d_ and a negative x-value indicates a preference for P_c_. Retrospective responses from subject 15 are missing. Note that the EMA overall in situ preference is calculated as the average rating for EMA questions “Sound quality”, “Speech understanding”, and “Program suitability” while “Loudness” was not considered.

Figure 4 displays the overall in situ preference as difference scores (i.e., mean preference for P_d_ minus mean preference for P_c_) on the *x*-axis against the retrospective preference on the *y*-axis. The in situ preference was slightly higher for P_d_ (mean difference = 0.14, SD = 1.06), albeit not significantly different (Wilcoxon paired signed-rank test, z = −1.33, *p* = 0.18). Moreover, “Loudness” ratings were higher for P_c_ than for P_d_ (mean difference = −0.47, SD = 1.13), but this difference was also not significant (Wilcoxon paired signed-rank test, z = 1.73, *p* = 0.08).

Notably, the in situ overall preference aligned well with the retrospective preference reports. However, four subjects (4, 6, 8, and 14) did not exhibit a clear in situ preference compared to in the retrospective reports, and one subject (9) exhibited a slightly higher overall in situ preference for P_d_ while preferring P_c_ at the retrospective follow-up questionnaire.

### 3.3. Insights from Volume Control Logs

We hypothesized that volume control logging reveals behavioral insights in support of the EMA evaluation of listening experiences with the BCD listening programs. Thus, we investigated whether volume control differed among the two listening programs in terms of overall level usage and level changes.

**Figure 5:**
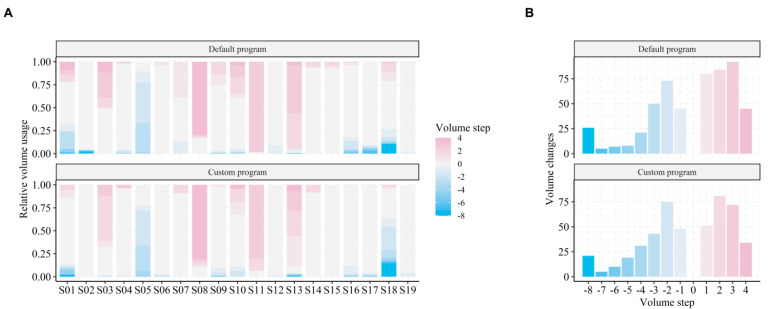
Volume level usage and level changes. (**A**) Relative volume usage for each listening program (panels) and each subject. (**B**) Number of volume changes made to each volume step across all subjects. The default volume (0) was activated on each device reset and program change. Thus, all changes to level 0 were not considered.

The volume control varied considerably among subjects and listening programs (see Figure 5A). Some subjects did not use volume control at all (e.g., subject 6), whereas others preferred to generally use lower (e.g., subject 5) or higher (e.g., subject 13) volume levels compared to the default setting (0 dB volume setting). In addition, more volume changes to levels above 0 were made for P_d_ (see Figure 5B). In total, 1026 volume adjustments (mean = 54, SD = 71) were made during the EMA trial period.

To quantify volume control, a linear-mixed effect (LME) model was applied to predict volume level changes from listening program and acoustic data. This tested whether subjects actively used the volume control (i.e., reacted to changes in the listening environments) and whether this behavior could potentially differentiate between the two listening programs. The applied LME model is described by:

(2) $$\mathrm{volume}=\mathrm{intercept}+\beta_{1}*\mathrm{SPL}+\beta_{2}*\mathrm{SNR}+\beta_{3}*\mathrm{program}+\gamma_{1}*\mathrm{ID}+\gamma_{2}*\mathrm{hour},$$

with fixed effect regression coefficients, $\beta_{1}$ to $\beta_{3}$, and random effects offsets $\gamma_{1}$ and $\gamma_{2}$. Note that the acoustic predictors SPL and SNR were centered and scaled prior to modeling, which means that the intercept predicts the volume for the observed grand mean SPL and SNR.

The regression coefficients in Table 3 show that volume changes were overall 0.74 step lower for P_c_ compared to P_d_ when evaluated at the mean observed levels of SNR and SPL. In addition, subjects preferred a lower volume when SPL increased, but a higher volume when SNR increased. A significant interaction between SPL and listening program indicates that this volume sensitivity was more pronounced with P_c_ than with P_d_. This pattern was not driven by SPL and SNR being inversely correlated, since variance inflation factors post modeling were low (2.2 for SPL and 2.9 for SNR). Instead, the pattern indicates that ambient SPL (e.g., related to loudness perception) and ambient SNR (e.g., related to signal perception) modulated changes in volume preference, and that subjects were more sensitive towards change in SPL when using the high-frequency gain program.

The LME model explained 48.8% of the variance in all volume changes. Out of this, 3.7% of the variance was contributed by the fixed effects (SPL, SNR and listening program) alone, whereas individual differences (i.e., different average volume per subject) captured 43.8% of the total explained variance. The remaining explained variance was captured by hourly fluctuations in volume changes.

### 3.4. Statistical Modeling of In Situ Reports

Average in situ ratings (Figure 4) and retrospective APHAB and SSQ scores did not reveal any significant differences in listening experience with the two listening programs. However, as highlighted in the previous section and shown in Figure 2, Figure 3, Figure 4 and Figure 5, inter-individual (e.g., device usage, volume control) and contextual factors (e.g., time of day, sound environments) contributed to variance in EMA ratings. Thus, averaging across time, subjects, and listening environments might mask differences in listening experiences with the two listening programs. Therefore, in this section, EMA ratings are assessed while considering contextual factors.

**Figure 6:**
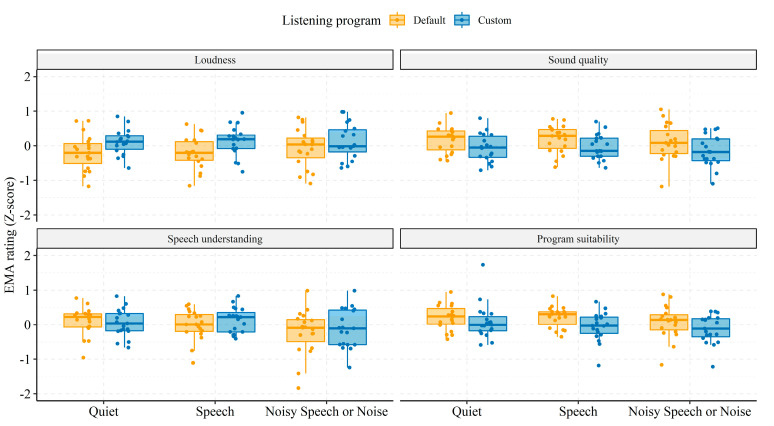
Boxplots of the average EMA rating for each question (panels) and listening program (colors) separated by soundscape (horizontal axis). EMA ratings were centered and scaled for each subject prior to averaging.

In Figure 6, boxplots of the average EMAs for each subject (individually scaled and centered) are shown stratified by soundscape and listening program. Across soundscapes, P_c_ was rated higher than P_d_ on “Loudness” (P_c_: M = 0.17, 95% CI = 0.15; P_d_: M = −0.16, 95% CI = 0.13), but lower on the “Sound quality” (P_c_: M = −0.02, 95% CI = 0.15; P_d_: M = 0.16, 95% CI = 0.11) and “Program suitability” (P_c_: M = −0.03, 95% CI = 0.12; P_d_: M = −0.17, 95% CI = 0.10) questions.

For the “Speech understanding” question, P_c_ was again rated higher than P_d_ but only for Quiet and Noisy Speech or Noise soundscapes. For the Speech soundscape, “Speech understanding” was rated slightly higher with P_c_ than P_d_ (P_c_: M = 0.13, 95% CI = 0.16; P_d_: M = 0.00, 95% CI = 0.19).

To allow for statistical testing and targeted context-dependent evaluation of in situ listening preferences (Figure 6), individual ratings of each EMA question were modelled using linear-mixed effect (LME) models, adjusted for variance due to inter-individual baseline offsets and sensitivity towards soundscapes, time-of-day, days since study start, and volume setting. Another advantage of using LME models for EMA data is that the modelling outcomes are appropriately weighted by sample sizes, which differed among the subjects (Table 2) and listening conditions (Figure 2).

The fixed-effects independent variables for LME modelling were listening program (P_d_ vs. P_c_), soundscape, and the interaction between the two. The associated regression coefficients are presented in Table 4.

At baseline levels, the marginal effects indicate that “Program suitability” ratings were higher in the Speech compared to the Quiet soundscape with P_d_. In addition, ratings of “Loudness” were higher with P_c_ than with P_d_ in the Quiet (baseline) soundscape. The significant interactions between soundscape and listening program suggest that “Sound quality”, “Speech understanding”, and “Program suitability” were all rated lower with P_c_ than with P_d_ in Noisy Speech or Noise soundscapes. In addition, the higher rating of “Loudness” with P_c_ was also present in Speech soundscapes but not in Noisy Speech or Noise soundscapes.

In summary, LME modeling revealed that the additional high-frequency gain in the custom listening program yielded higher ratings of “Loudness” for Quiet and Speech soundscapes and lower ratings of “Sound quality”, “Speech understanding”, and “Program suitability” for the more challenging Speech in Noise and Noise soundscapes.

We also checked whether adjusting for volume, time, and day in the LME models was warranted. That is, we assessed whether these contextual factors affected how ratings were performed in the trial period. Each fitted model was compared to more simple models that only adjusted for inter-individual differences in EMAs. In all cases, adding the additional contextual parameters as co-regressors significantly improved model fits when testing with likelihood ratio tests (Loudness: dAIC = 10, Chi2(2) = 16.10, *p* = 0.001; Sound quality: dAIC = 74, Chi2(2) = 79.88, *p* < 0.001; Speech understanding dAIC = 60, Chi2(2) = 66.37, *p* < 0.001; Program suitability dAIC = 49, Chi2(2) = 55.10 *p* < 0.001).

## 4. Discussion

Differences in listening abilities and experiences with two BCD listening programs were investigated in a heterogeneous sample of 19 unilaterally fitted subjects. The listening programs differed only in high-frequency gain. Laboratory measures of speech reception thresholds revealed a significant benefit of the additional high-frequency gain in the speech-in-quiet condition, but not for the speech-in-noise condition. Moreover, retrospective preference ratings with the SSQ and APHAB questionnaires did not reveal significant differences in listening experiences with the two listening programs. However, Ecological Momentary Assessments (EMAs) combined with data logging of the auditory ecology of subjects identified distinct patterns in listening experiences. The additional high-frequency gain resulted in higher “Loudness” ratings in quiet and pure speech listening environments. On the other hand, the default prescribed listening program was rated higher on “Sound quality”, “Speech understanding”, and “Program suitability” when in noisy speech or noise conditions. These findings suggest that additional high-frequency gain can be beneficial for speech understanding, but only in quiet and ideal listening conditions driven by an increased audibility (i.e., higher “Loudness” rating). In fact, EMA data indicated that additional high-frequency gain resulted in real-world listening experiences that were judged as too loud, even for the pure speech soundscape. Thus, the data do not provide evidence for the hypothesized benefit in speech recognition from the availability of more high-frequency speech cues [9,43,45]. Instead, the results suggest that real-life experiences should be considered more closely when fitting high-frequency amplification in BCD users.

Subjects tended to use the volume control differently with the two programs and used overall lower levels of volume with the custom gain program, most likely to compensate for the additional high-frequency gain. By analyzing volume control logs, we were able to identify global patterns of volume control. Specifically, we found that subjects preferred to change to higher levels of volume when in optimal listening environments (i.e., high SNR), whereas lower levels of volume were activated when the sound pressure levels increased and vice versa. In addition, volume preferences depended on the listening program, with higher volumes being preferred for the default compared to the custom high-frequency gain program. This corroborates well with the documented increase in loudness perception when using the high-frequency gain program. However, strong inter-individual differences in volume control were evident. That is, some subjects consistently changed to higher or lower volume levels than the default level (see Figure 5, e.g., subject 5 and 8), independent of listening program. The inter-individual differences in volume control were not explained by HL characteristics (Table 1). Instead, abnormal volume control (i.e., more often choosing a level different than the default) might indicate an insufficient fitting—that is, a fitting with consistently too little or too much gain according to an individual’s needs and listening preferences.

A possible explanation for the discrepancy between benefits of custom high-frequency gain in the laboratory versus in real-world could be because of the difference in testing hearing at threshold levels (i.e., small or negative SNRs and low SPLs) and relating listening experiences to supra-threshold listening conditions (i.e., real-world conditions). The documented SRTs, which were statistically different between the two listening programs, were 39.1 dB SPL for P_c_ and 36.9 dB SPL for P_d_. These levels are much lower than the ones faced in real life [34,44], and lower than the levels associated with EMA submissions in the current study (See Figure 2). In addition, the encountered real-world SNRs vastly differ from those during laboratory testing. In fact, only 6.8% of the recorded SNRs during normal BCD usage in the current study had levels less than zero, and the 25% and 75% quantiles were 1.5- and 10.4-dB SNR, respectively. Furthermore, the subjects encountered slightly higher SNRs when performing EMAs compared to periods of normal BCD usage (median SNR when performing EMA was 7.5 dB, and 4.3 dB for normal BCD usage). Thus, the additional high-frequency gain expected to aid in speech perception was most likely not necessary in everyday usage, since SNRs were favorable.

A possible solution for increasing real-world speech perception by increasing high-frequency gain would be to implement fast acting compression on the acoustic sources and take the ambient SNR into account [46]. Compression in the custom high-frequency gain program would have reduced high-level gain differences between both listening programs, and it would have reduced effects of device saturation for high-level inputs.

The relatively high real-world SNRs documented in our study are comparable to those found in previous studies [20,22,23]. This indicates that despite differences in recording equipment, SNR estimates in the current study derived directly from the BCDs are valid. In addition, the distributions of EMAs per soundscape and time of day (Figure 2B) are comparable to those previously found in studies using the EMA methodology to assess behind-the-ear (BTE) hearing aid benefits [35]. This suggests that the distributions are shaped from normal device usage patterns (e.g., Speech in Noise soundscapes naturally occurring more often during noon/midday activities), and that usage patterns are similar among BTE and BCD users. The current study therefore validates the use of EMAs for evaluating listening experiences in BCD users and expands upon previous work by including behavioral insights from data logging to support the EMA outcomes. Crucially, data logging ensures that confounding factors can be adjusted for when modeling the EMA reports. For example, ecological behavior will entail user changes in volume control (e.g., Figure 5), which if not adjusted for, will skew the EMA modeling outcomes and produce worse model fits.

Besides providing evidence of everyday listening experiences, the current study shows that EMA data can help track day-to-day acclimatization to hearing device features. None of the subjects in the current study had prior experience with the Ponto 4 BCD, and a certain acclimatization would therefore be expected [29]. Figure 3 shows that acclimatization (i.e., change in EMA rating per day) is especially pronounced in questions of “Sound quality” and suitability of the listening program, whereas there were no significant effects of acclimatization in ratings of “Loudness” or “Speech understanding”. Thus, retrospective questionnaires targeting multiple dimensions of listening experiences will inevitably be differently affected by the level of acclimatization achieved and the time point at which such questionnaires are given. Moreover, as the test-retest reliability of EMAs has been found to be high [47], any acclimatization effect identified by day-to-day changes in EMAs reflects real changes in listening experiences.

In summary, our current findings highlight the potential of supporting traditional outcome measures of assistive hearing technology with ecologically valid EMA and real-world data logging. Outcome measures from laboratory tests typically target threshold hearing abilities under artificial conditions, whereas real-world EMA and data logging assess both on-the-spot subjective listening experiences and objective dimensions of hearing health behavior (e.g., sound exposure, volume/program control, device usage times), which are important for interpretation of EMA ratings.

## 5. Limitations

### 5.1. Representativeness of In Situ Reports of Listening Experience

Ideally, and to ensure representativeness, EMAs should be completed in varying listening conditions throughout the day. However, assessments are likely biased, since the smartphone used to complete them is not carried along in all possible listening environments [48]. In the current study, significant results were obtained for both Speech and Noisy Speech or Speech soundscapes, which suggests that sampling was sufficient under those listening conditions. Alternatively, and to increase statistical power, the EMA protocol could include a prompt to perform EMA triggered by certain sound environments. This would ensure balanced sampling as recommended by [48]. On the other hand, such prompting might induce a burden on the subjects, and result in artificially forced ratings, which in the end might skew the EMAs to specific listening conditions. Instead, any prompting should occur with the hearing setting to be tested in mind. That is, if a noise reduction algorithm is being evaluated, noisy environments should predominantly trigger EMAs.

### 5.2. Relationship between EMA and Hearing Loss

The subjects were heterogeneous with respect to the aetiology of HL (e.g., PTA, laterality, BCD experience, see Table 1). However, we did not find that the PTA on the fitted side explained mean EMA ratings of listening programs (Pearson’s product-moment correlation, max r = 0.117, all *p* > 0.1). This was also true after stratifying the EMA data by soundscape. The limited sample size (i.e., *N* = 19) restricted further investigation into the effect of HL classification on listening experiences.

## 6. Conclusions

This novel approach to evaluating BCD performance opens up interesting possibilities for optimizing device settings by combining real-life in situ subjective experiences with listening environments and device parameters. Additionally, experiences and data logs from ecologically relevant situations can be used as a basis for in-depth counselling [49]. We find that despite being better in laboratory tests of speech perception in quiet conditions, custom high-frequency gain leads to real-world listening experiences that are rated too loud in speech- or noise-dominated sound environments. Logging of volume control suggests that subjects compensated for the additional high-frequency gain by lowering volume settings in high intensity and noisy environments despite large inter-individual differences in volume control. In summary, this study shows that, on average, the default gain prescription is appropriate for most subjects, with some notable exceptions that cannot be explained by HL characteristics. It also shows that EMA ratings for “Sound quality” and suitability of listening program may be used to monitor acclimatization to differences in gain settings or new hearing devices.

## Figures and Tables

**Table 1:** Subject characteristics. Columns from left to right: subject, age in years, sex, experience with BCD (years; month), aetiology of hearing impairment for left (AS) and right (AD) side (NH: normal hearing; CSOM: chronic suppurative otitis media; Atresia: congenital atresia of the ear canal; SN: sensorineural loss; Presb: Presbycusis), pure-tone average thresholds for left and right side at 0.5, 1, 2, 4 kHz for air conduction (AC) and bone conduction (BC), fitted side, and usage of the listening programs during the 4-week trial period (default program: Pd, custom program Pc) in hours/day. Most subjects had about 5-years of BCD experience, except for subjects 8 (2 months) and 12 (4 months). Usage time was recorded directly from the BCD. Subjects 1 and 15 did not switch off their BCD but used standby when not in use.

Subject	Age (years)	Sex	Exp (year; month)	Aetiology		PTA_0.5,1,2,4_ (dB)				Fitted Side	Usage (hr/day)	
						AS		AD				
				AS	AD	AC	BC	AC	BC		P_d_	P_c_
1	42	F	4; 9	CSOM	CSOM	47.5	20.0	73.8	16.3	AD	16.1 ^1^	1.8
2	30	M	4; 7	NH	CSOM	6.3	2.5	35.0	7.5	AD	8.3	3.8
3	54	F	4; 11	CSOM	CSOM	58.8	3.8	48.8	6.3	AS	12.2	4.1
4	72	M	5; 0	CSOM	CSOM	81.3	31.3	66.3	30.0	AD	8.3	6.2
5	47	M	5; 7	Atresia	Atresia	68.8	16.3	70.0	16.3	AD	4.5	13.0
6	47	M	5; 3	CSOM	SN	92.5	33.8	31.3	23.8	AS	3.8	2.5
7	70	M	4; 9	Presb	CSOM	25.0	25.0	82.5	36.3	AD	7.8	7.3
8	53	F	0; 2	CSOM	CSOM	52.5	22.5	52.5	32.5	AD	9.3	4.0
9	65	M	7; 6	CSOM	CSOM	43.8	15.0	71.3	30.0	AS	6.7	6.8
10	34	M	6; 2	CSOM	CSOM	78.8	18.8	40.0	12.5	AS	7.7	5.1
11	59	M	6; 4	CSOM	CSOM	72.5	33.8	51.3	27.5	AS	11.5	3.3
12	46	F	0; 4	CSOM	NH	56.3	17.5	5.0	2.5	AS	9.3	3.0
13	47	F	5; 9	Atresia	Atresia	75.0	17.5	72.5	17.5	AD	4.7	10.0
14	56	F	5; 5	CSOM	CSOM	66.3	23.8	67.5	36.3	AD	15.7	1.8
15	27	F	5; 5	CSOM	CSOM	57.5	11.3	41.3	27.5	AD	18.3 ^1^	5.2
16	19	M	5; 6	NH	Atresia	10.0	−2.5	61.3	6.3	AD	2.1	2.6
17	51	M	4; 9	Deaf	CSOM	* ^2^	* ^2^	50.0	20.0	AD	6.5	5.3
18	57	F	4; 7	CSOM	CSOM	65.0	21.3	41.3	12.5	AS	8.3	4.5
19	22	M	4; 7	NH	Atresia	6.3	8.8	65.0	7.5	AD	9.2	2.8

^1^ BCD on standby when not in use; ^2^ * unmeasurable threshold.

**Table 2:** Data logging summary. Columns from left to right: subject, logged usage for both the default listening program (P_d_) and the custom program (P_c_) in hours per day, the absolute number of submitted EMA’s per listening program, the proportion of submitted EMAs for the soundscapes Quiet, Speech, Noisy speech or noise, and usage logged on the smartphone divided by total device uptime (i.e., proportion of device uptime with smartphone connection).

Subject	Days	Logged Usage (hour/day)		Submitted EMAs (Count)		Proportion of Logged Usage and Submitted EMAs per Soundscape (%-Usage; %-EMA)			Total Logged Usage/Total Wear Time (%)
		P_d_	P_c_	P_d_	P_c_	Quiet	Speech	Noisy Speech or Noise	
1	22	0.80	1.86	25	40	38; 46	33; 37	29; 17	15
2	26	1.92	1.29	35	41	24; 41	29; 26	48; 33	27
3	24	1.44	1.79	22	21	29; 49	38; 28	33; 23	20
4	34	4.88	3.25	69	66	23; 41	37; 41	40; 19	56
5	34	10.63	3.08	175	151	23; 29	33; 36	44; 34	78
6	27	1.94	1.73	94	73	46; 54	27; 23	27; 23	58
7	24	0.58	0.96	28	31	23; 37	53; 46	24; 17	10
8	27	6.82	6.23	89	73	57; 71	29; 23	14; 6	98
9	27	5.59	3.72	200	188	50; 59	38; 31	12; 10	69
10	24	2.62	3.07	93	78	18; 33	35; 23	48; 44	44
11	30	3.02	8.41	74	83	38; 66	49; 31	13; 3	77
12	14	2.92	1.48	20	14	44; 74	45; 18	12; 9	36
13	28	7.13	2.41	208	174	41; 54	31; 23	28; 23	65
14	18	1.98	2.16	16	11	39; 37	37; 37	23; 26	24
15	21	5.72	4.61	28	34	69; 58	24; 32	7; 10	44
16	31	2.44	1.24	43	41	12; 20	51; 38	37; 42	78
17	29	2.10	3.79	41	45	45; 50	42; 40	14; 10	50
18	29	5.06	3.71	89	85	39; 41	45; 45	16; 14	69
19	30	3.70	2.10	89	58	26; 34	44; 37	30; 29	48
Mean	26.3	3.75	2.99	68.79	75.68	36; 47	38; 32	26; 21	49
SD	5.0	2.57	1.87	51.10	59.92	14; 15	8; 8	13; 12	24

**Table 3:** Regression coefficients (β) with 95% CIs for predicting volume control changes. The predictors were ambient sound environment characteristics (SPL and SNR, scaled and centered) and listening program (*n* = 1026). The default listening program was used for baseline contrast.

Coefficient	β	95% CI
SPL	−0.67 **	−0.92 to −0.43
SNR	0.30 **	+0.08 to +0.52
P_c_	−0.74 **	−1.04 to −0.44
SPL:P_c_	0.34 *	+0.00 to +0.70
SNR:P_c_	−0.04	−0.27 to +0.36
Intercept	0.09	−0.97 to +1.14

Note: SPL = sound pressure level; SNR = signal-to-noise ratio; P_c_ = custom listening program; “:” indicates interaction; * *p* < 0.05; ** *p* < 0.01. Baseline listening program = P_d_ (default listening program).

**Table 4:** Regression coefficients (β) with 95% CIs from LME models predicting EMA ratings. Baseline contrasts were Quiet soundscape and the default listening program.

Coefficient	Loudness	Sound Quality	Speech Understanding	Program Suitability
	β (95% CI)	β (95% CI)	β (95% CI)	β (95% CI)
S	−0.10 (+0.28 to +0.08)	0.03 (−0.17 to +0.24)	0.05 (−0.16 to +0.26)	0.18 * (+0.00 to +0.36)
NSoN	0.20 (−0.06 to +0.46)	0.01 (−0.33 to +0.34)	−0.20 (−0.53 to +0.14)	0.11 (−0.20 to +0.41)
P_c_	0.42 ** (+0.28 to +0.55)	−0.14 (−0.29 to +0.01)	0.05 (−0.13 to +0.23)	−0.11 (−0.26 to +0.04)
S:P_c_	0.34 ** (+0.12 to +0.55)	−0.10 (−0.34 to +0.14)	−0.01 (−0.29 to +0.26)	−0.22 (−0.46 to +0.02)
NSoN:P_c_	−0.01 (−0.25 to +0.23)	−0.40 ** (−0.66 to −0.13)	−0.47 ** (−0.78 to −0.16)	−0.66 ** (−0.93 to −0.39)
Intercept	4.75 (+4.09 to +5.41)	6.78 (+6.22 to +7.34)	7.05 (+6.40 to +7.69)	6.77 (+6.17 to +7.37)
Observations	2745	2745	2323	2745
Expl. Variance	62.6%	57.1%	55.4%	61.7%

Note: S = Speech; NSoN = Noisy Speech or Noise; P_c_ = custom listening program; “:” indicates interaction; * *p* < 0.05; ** *p* < 0.01. Baseline listening program = P_d_ (default listening program) and baseline soundscape = Quiet.
